# CACNA1B gene variants in adult-onset isolated focal dystonia

**DOI:** 10.1007/s10072-020-04778-8

**Published:** 2020-10-13

**Authors:** Relu Cocoș, Florina Raicu, Ovidiu Lucian Băjenaru, Iulia Olaru, Laura Dumitrescu, Bogdan Ovidiu Popescu

**Affiliations:** 1 Department of Medical Genetics, Carol Davila University of Medicine and Pharmacy, 37 Dionisie Lupu Str, 020021 Bucharest, Romania; 2 Francisc I. Rainer Anthropological Research Institute, Romanian Academy, 8 Eroii Sanitari Bld, 050474 Bucharest, Romania; 3 Department of Clinical Neurosciences, Colentina Hospital Neurology Division, Carol Davila University of Medicine and Pharmacy, 37 Dionisie Lupu Str, 020021 Bucharest, Romania; 4 Ana Aslan National Institute of Geriatrics and Gerontology, 9 Căldărușani Str, 011241 Bucharest, Romania; 5 Department of Neurology, Colentina Clinical Hospital, 19-21 Stefan cel Mare Str, 020125 Bucharest, Romania; 6 Laboratory of Ultrastructural Pathology, Victor Babeș National Institute of Pathology, 99-101 Splaiul Independentei Str, 050096 Bucharest, Romania

**Keywords:** CACNA1B gene, Disease-causing variants, Isolated focal dystonia, Next-generation sequencing

## Abstract

**Background:**

Isolated focal dystonia (IFD) is a heterogeneous group of potentially invalidating movement disorders. The etiopathogenesis is complex, both genetic and environmental factors playing a role, but remains elusive. The CACNA1B gene codes for the N-type neuronal voltage-gated calcium channels CaV2.2, which may play a role in the development of some IFD.

**Methods:**

We analyzed samples from the GENDYS cohort for mutations in CACNA1B gene, using targeted next-generation sequencing (NGS).

**Results:**

The GENDYS cohort consists of 120 people with adult-onset IFD (cervical dystonia 47.5%, blepharospasm 47.2%, others 8.3%). Of these, 35% had subsequent topographical extension. Average age at onset was 42 and average disease durations 8 years. Targeted NGS revealed a novel frameshift mutation c.2291AGG > A, in exon 19, and a previously reported variant, c.6834T > G, in exon 47.

**Conclusion:**

Our findings suggest that disease-causing mutations in CACNA1B gene may be involved in the development of some adult-onset IFD. To our knowledge, this is the first study that identified a disease-causing CACNA1B gene mutation in association with adult-onset IFD.

## Introduction

Isolated dystonia is a heterogeneous group of under-diagnosed movement disorders [1]. The main clinical findings are the dystonic movements and postures, defined by “sustained or intermittent muscle contractions causing abnormal, often repetitive, movements, postures, or both” [1, 2]. Depending on the body distribution, isolated dystonia is classifiable as focal, segmental, multifocal, generalized, and unilateral [2]. In those with adult-onset, the most common form is isolated focal dystonia (IFD), torticollis, and blepharospasm being the typical phenotypes, sometimes with subsequent progression to other regions [1].

Since the discovery that mutations in the TOR1A gene (i.e., DYT1) are responsible for most of the early-onset isolated dystonia cases, a growing number of genes linked to Mendelian forms of dystonia have been identified [3]. The etiopathogenesis of adult-onset dystonia is known to a lesser degree, both genetic predisposition and environmental factors probably playing a role [2, 3].

To date, a total of 26 loci symbolized as DYTs (i.e., DYT1 to DYT25) are known to be involved in dystonia syndromes [4]. Of these, three causative genes have been extensively validated in different populations, namely TOR1A (DYT1), THAP1 (DYT6), and GNAL (DYT25) [1, 4]. Other genes found to be associated with isolated or combined dystonia include ANO3 (DYT24), TUBB4A (DYT4), GCH1 (DYT5a), TH (DYT5b), TAF1 (DYT3), PRKRA (DYT16), ATP1A3 (DYT12), SGCE (DYT11), KCTD17, and CACNA1A [4–8].

The CACNA1B gene (chromosome 9, q34.3) codes for the pore-forming α1B subunit of the Ca_V_2.2 N-type voltage-gated calcium channels, which are essential for neurotransmitter release from cerebral neurons [6, 9, 10]. Common variants in CACNA1B were linked to the risk of cerebral infarction [9], bipolar disorders [10], and schizophrenia [10, 11]. Recently, CACNA1B mutations were also found in association to a particular myoclonus-dystonia syndrome [12] and progressive epilepsy-dyskinesia [13]. The former association was not replicated in a large European cohort, but the study focused only on the previously reported R1389H variant, the CACNA1B gene not being extensively screened [14]. Targeted next-generation sequencing (NGS) can help identify and investigate genes with a great number of exons, such as CACNA1B. Considering its putative role in some primary dystonia syndromes, we screened the CACNA1B gene for disease-causing variants using targeted NGS Ion Torrent PGM in a large cohort of patients with adult-onset IFD. To our knowledge, this is the first study that used targeted NGS to identify potentially predisposing variants in CACNA1B gene.

## Methods

The research was conducted in accordance with the Helsinki declaration, with approval from the Research Ethics Committee at Colentina Clinical Hospital (Bucharest, Romania), as part of a more extensive project on the genetics of adult-onset IFD (i.e., GENDYS-UEFISCDI, PN-III-P4-ID-PCE-2016-0696). All patients signed a dedicated written informed consent prior to enrolment.

### Patients and DNA extraction

The present work is based on the GENDYS cohort that consists of 120 people with adult-onset IFD (36 males and 84 females), with or without subsequent topographical extension. Of these, most have torticollis and other cervical dystonia syndromes (i.e., 47.5%) and blepharospasm (47.2%)—for further details, please see Table 1. The cohort was recruited from patients diagnosed and treated at Colentina Clinical Hospital, based on predefined inclusion and exclusion criteria. The IFD diagnosis was made by a senior neurologist with international expertise in the field, in agreement with current guidelines and consensus [1, 2, 15]. In order to minimize ethnicity-related genetic findings, only patients affirming Romanian descent were included.

**Table 1:** Demographic and phenotypic characteristics of the GENDYS IFD cohort

Characteristics/phenotypes	Cervical dystonia	Blepharospasm	Other IFD	All IFD
Total number (%)	57 (47.5%)	53 (47.2%)	10 (8.3%)	120
Male (%)	38 (66.7%)	41 (77.4%)	5 (50%)	36 (30%)
Female (%)	19 (33.3%)	12 (22.6%)	5 (50%)	84 (70%)
Average age at onset (years)	42	53	34	46
Disease duration (years)	8	8	8	8
Number with progression to other regions	5 (8.8%)	26 (49.1%)	4 (40%)	35 (29.2%)

Oral mucosa swabs were collected from all patients enrolled in the GENDYS cohort. Genomic deoxyribonucleic acid (DNA) was extracted from the oral mucosa cells using the *PureLink Genomic DNA kit* according to the protocol of the manufacturer. Prior to analysis, the genomic DNA concentration was quantified by *Qubit dsDNA BR Assay kit*, and the DNA integrity was checked by agarose gel.

### Library preparation, emulsion PCR, and sequencing

The primers for target region comprising all coding exons, intron-exon boundaries flanking sequences (padding + 10 base pairs), and untranslated regions (UTR) of CACNA1B gene were automatically generated by *Ion AmpliSeq designer software*. Library preparation was performed using the *Ion AmpliSeq Library kit 2.0* (Thermo Fisher Scientific Inc. TM, Waltham, USA) according to the manufacturer’s procedures. Ten nanograms of gDNA was used for library preparation. DNA was amplified with 21 amplification cycles using primers and *AmpliSeq HiFi mix*.

Following polymerase chain reaction (PCR) amplicon digestion with *FuPa reagent*, the libraries were indexed using the *Ion Xpress Barcode Adapter 1-16 Kit*. The libraries were then purified using the *Agencourt AMPure XP Reagent* (Beckman Coulter Genomics, Danvers, MA, USA). The concentration of the final libraries was quantified by fluorescent measurement on Qubit 2.0 instrument using the *QubitTM dsDNA HS Assay Kit* (Life Technologies, CA, USA). After quantification, the amplicon libraries were diluted to 100 pM.

A maximum of 16 amplicon libraries were pooled for emulsion PCR (ePCR) on an Ion OneTouch2 System using the *Ion PGM HI-Q View OT2 Kit.* Following ePCR, template positive ion sphere particles (ISP) were enriched using the Ion OneTouch ES (Thermo Fisher). Sequencing of the template positive ion sphere particles was performed on the Ion PGM with *Ion 318v2 BC Chip* and the *Ion PGM Hi-Q View Sequencing kit* (Life Technologies, CA, USA) using 500 flows.

Sequence variants were confirmed by resequencing the selected samples using targeted NGS.

### Bioinformatics analysis

Raw sequence data analysis was performed using *Torrent Suite Software v.5.6* (Life Technologies), to generate good quality reads by base calling, trimming adapter and primer sequences, and filtering out low-quality reads. The reads were demultiplexed according to the barcode sequence.

The ion torrent sequence data were than aligned and mapped to the hg19/GRCh37 human reference genome using the Torrent Mapping Alignment Program using the default parameters. The generated BAM files with aligned reads were processed using Variant Caller plugin included in the Torrent Suite Variant Caller TVC program.

Called variants aligned to the reference genome were visualized using Tablet (James Hutton Institute, Scotland, UK) [16] to view the rescaled binary alignment map (BAM) files in order to confirm the variant calls and check the discordant results. Following variant calling, the variants in Variant Calling Format file were annotated with ANNOVAR. The scripts included in ANNOVAR allowed to include in the analysis multiple public genomic database such as ClinVar, dbSNP, Exome Variant Server (esp6500), Exome Aggregation Consort (ExAC), and 1000 Genomes Project.

We compared all detected variants using human gene mutation database (HGMD) and predicted the effect of identified variants on the protein function by three in silico prediction software PolyPhen-2 [17], Mutation Taster [18], and SIFT [19].

## Results

**Fig. 1:**
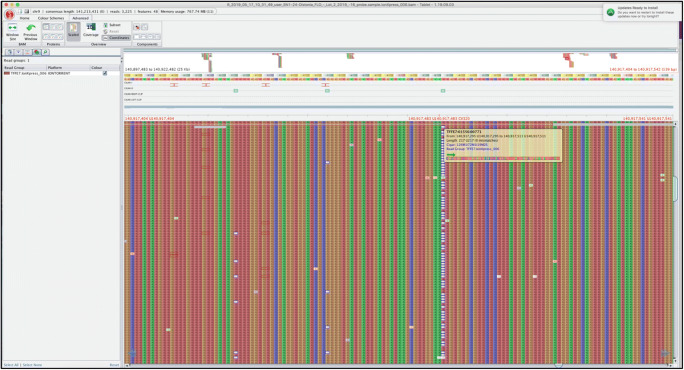
CACNA1B sequencing reads viewed using Tablet software showing the c.2291AGG > A mutation

**Fig. 2:**
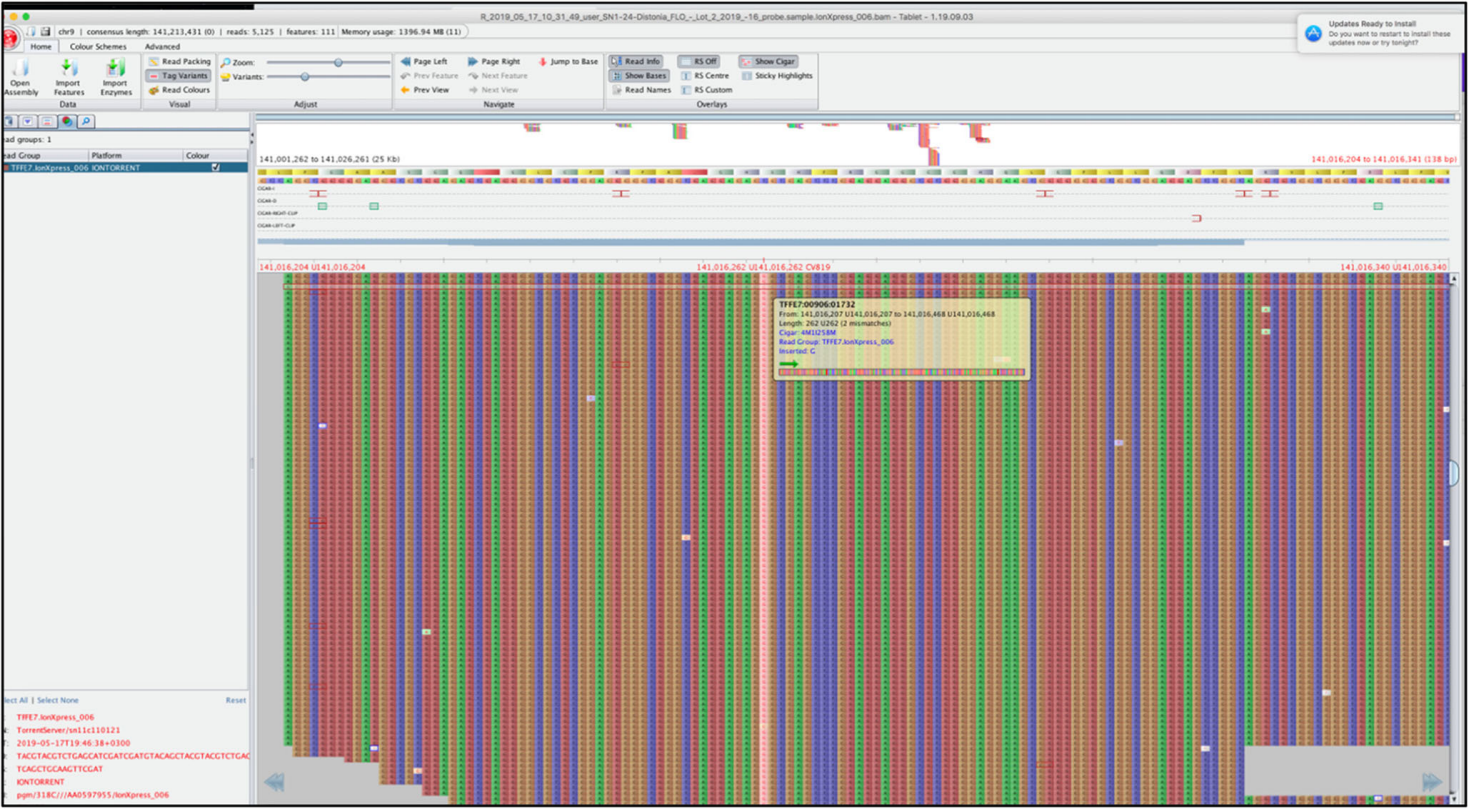
CACNA1B sequencing reads viewed using Tablet software showing the c.6834T > G variant

We analyzed samples from 120 adult-onset IFD patients for mutations in CACNA1B gene, using targeted NGS. Amplicons ranged from 120 to 310 pb, with a mean amplicon length of 237 pb and at least 110-fold depth covering 100% of the bases of the targeted gene. The sequence analysis by NGS revealed a novel frameshift mutation c.2291AGG > A (Fig. 1), in exon 19, and a previously reported variant, c.6834T > G (Fig. 2), in exon 47. The mutation/variants are named according to the nomenclature of Human Genome Variation Society. Segregation analyses were not performed for the mutation because paternal DNA was unavailable for testing. The mutation was not found in 120 unrelated control individuals from the same ethnic origin.

For predicting the impact of the novel frameshift mutation, we used three in silico programs: PolyPhene-2, SIFT, and Mutation Taster PolyPhene 2, damaging; SIFT, damaging; Mutation Taster, disease causing. The novel mutation was not reported in the control databases gnomAD, 1000 Genomes, and Exome Variant Server or in any other single-nucleotide polymorphism database. The online software tools predict that c.6834T > G causes the deleterious leucine acid to arginine substitution at position 2215, pLeu2215Arg (PolyPhene 2, unknown; SIFT, tolerated; Mutation Taster, disease causing) in one of two isoforms of CACNA1B. The c.6834T > G variant is predicted as “disease causing” by Mutation Taster, but it can be classified as likely benign, considering that allele frequency is greater than that expected for a disorder. The two variants/mutation were found recurrently; thus, c.6834T > G pLeu2215Arg (rs2278973) was detected in 38 (31.7%) of our 120 IFD patients in homozygous and heterozygous state, and the novel c.2291AGG > A frameshift mutation was present in 6 (5%) unrelated cases in heterozygous state.

## Discussion

The CACNA1B gene codes for the N-type neuronal voltage-gated calcium channels CaV2.2, which may play a role in the development of some movement disorders, including IFD. Prior to our study, genetic analysis of the CACNA1B gene in patients with movement disorder was performed in a limited number of studies, by methods not suited for wide gene screening. Mutations were found only in patients with myoclonus-dystonia, a rare form of combined dystonia [3, 12]. The targeted NGS approach allowed us to quickly screen for disease-causing variants of CACNA1B gene in a cohort of 120 patients with adult-onset IFD. We identified a novel frameshift mutation (i.e., c.2291AGG > A) that may have damaging effects on the CaV2.2 N-type voltage-gated calcium channels. To our knowledge, this is the first study that identifies a disease-causing mutation with loss of function effect in the CACNA1B gene association with adult-onset IFD.

## Conclusion

Considering the lack of this mutation in the control group and the putative pathogenicity estimated by the in silico prediction tools, our results support a causal association between c.2291AGG > A variant and IFD. The effect of this frameshift mutation should be further analyzed by functional studies.

## Data Availability

Additional supplementary data are available per request.

## Abbreviations

ANO3: Anoctamin 3 (gene)
ANNOVAR: ANNOtate VARiation (bioinformatics software)
ATP1A3: ATPase Na+/K+ transporting subunit alpha 3 (gene)
CACNA1A: Calcium voltage-gated channel subunit alpha1 A (gene)
CACNA1B: Calcium voltage-gated channel subunit alpha1 B (gene)
DNA: Deoxyribonucleic acid
ePCR: Emulsion PCR
GCH1: GTP cyclohydrolase 1 (gene)
GNAL: G protein subunit alpha L (gene)
HGMD: Human Gene Mutation Database
IFD: Isolated focal dystonia
KCTD17: Potassium channel tetramerization domain containing 17 (gene)
NGS: Next-generation sequencing
PCR: Polymerase chain reaction
PRKRA: Protein activator of interferon-induced protein kinase EIF2AK2 (gene)
SGCE: Sarcoglycan epsilon (gene)
TAF1: TATA-box binding protein associated factor 1 (gene)
TH: Tyrosine hydroxylase (gene)
THAP1: THAP domain containing 1 (gene)
TOR1A: Torsin family 1 member A (gene)
TUBB4A: Tubulin beta 4A class IVa (gene)

## References

[1] Albanese A, Di Giovanni M, Lalli S (2019). Dystonia: diagnosis and management. Eur J Neurol.

[2] Albanese A, Bhatia K, Bressman SB, Delong MR, Fahn S, Fung VS, Hallett M, Jankovic J, Jinnah HA, Klein C, Lang AE, Mink JW, Teller JK (2013). Phenomenology and classification of dystonia: a consensus update. Mov Disord.

[3] Gonzalez-Alegre P (2019). Advances in molecular and cell biology of dystonia: focus on torsinA. Neurobiol Dis.

[4] Balint B, Bhatia KP (2015). Isolated and combined dystonia syndromes - an update on new genes and their phenotypes. Eur J Neurol.

[5] Marce-Grau A, Correa M, Vanegas MI, Munoz-Ruiz T, Ferrer-Aparicio S, Baide H, Macaya A, Perez-Duenas B (2019). Childhood onset progressive myoclonic dystonia due to a de novo KCTD17 splicing mutation. Parkinsonism Relat Disord.

[6] Jinnah HA, Sun YV (2019). Dystonia genes and their biological pathways. Neurobiol Dis.

[7] Tian J, Vemula SR, Xiao J, Valente EM, Defazio G, Petrucci S, Gigante AF, Rudzinska-Bar M, Wszolek ZK, Kennelly KD, Uitti RJ, van Gerpen JA, Hedera P, Trimble EJ, LeDoux MS (2018). Whole-exome sequencing for variant discovery in blepharospasm. Mol Genet Genomic Med.

[8] Montaut S, Tranchant C, Drouot N, Rudolf G, Guissart C, Tarabeux J, Stemmelen T, Velt A, Fourrage C, Nitschke P, Gerard B, Mandel JL, Koenig M, Chelly J, Anheim M, Ps F, Movement Disorders C (2018). Assessment of a targeted gene panel for identification of genes associated with movement disorders. JAMA Neurol.

[9] Yamaguchi M, Nakayama T, Fu Z, Sato N, Soma M, Morita A, Hinohara S, Doba N, Mizutani T (2010). The haplotype of the CACNA1B gene associated with cerebral infarction in a Japanese population. Hereditas.

[10] Curtis D, Vine AE, McQuillin A, Bass NJ, Pereira A, Kandaswamy R, Lawrence J, Anjorin A, Choudhury K, Datta SR, Puri V, Krasucki R, Pimm J, Thirumalai S, Quested D, Gurling HM (2011). Case-case genome-wide association analysis shows markers differentially associated with schizophrenia and bipolar disorder and implicates calcium channel genes. Psychiatr Genet.

[11] Glessner JT, Reilly MP, Kim CE, Takahashi N, Albano A, Hou C, Bradfield JP, Zhang H, Sleiman PM, Flory JH, Imielinski M, Frackelton EC, Chiavacci R, Thomas KA, Garris M, Otieno FG, Davidson M, Weiser M, Reichenberg A, Davis KL, Friedman JI, Cappola TP, Margulies KB, Rader DJ, Grant SF, Buxbaum JD, Gur RE, Hakonarson H (2010). Strong synaptic transmission impact by copy number variations in schizophrenia. Proc Natl Acad Sci U S A.

[12] Groen JL, Andrade A, Ritz K, Jalalzadeh H, Haagmans M, Bradley TE, Jongejan A, Verbeek DS, Nurnberg P, Denome S, Hennekam RC, Lipscombe D, Baas F, Tijssen MA (2015). CACNA1B mutation is linked to unique myoclonus-dystonia syndrome. Hum Mol Genet.

[13] Gorman KM, Meyer E, Grozeva D, Spinelli E, McTague A, Sanchis-Juan A, Carss KJ, Bryant E, Reich A, Schneider AL, Pressler RM, Simpson MA, Debelle GD, Wassmer E, Morton J, Sieciechowicz D, Jan-Kamsteeg E, Paciorkowski AR, King MD, Cross JH, Poduri A, Mefford HC, Scheffer IE, Haack TB, McCullagh G, Deciphering Developmental Disorders S, Consortium UK, BioResource N, Millichap JJ, Carvill GL, Clayton-Smith J, Maher ER, Raymond FL, Kurian MA (2019). Bi-allelic loss-of-function CACNA1B mutations in progressive epilepsy-dyskinesia. Am J Hum Genet.

[14] Mencacci NE, R'Bibo L, Bandres-Ciga S, Carecchio M, Zorzi G, Nardocci N, Garavaglia B, Batla A, Bhatia KP, Pittman AM, Hardy J, Weissbach A, Klein C, Gasser T, Lohmann E, Wood NW (2015). The CACNA1B R1389H variant is not associated with myoclonus-dystonia in a large European multicentric cohort. Hum Mol Genet.

[15] Albanese A, Asmus F, Bhatia KP, Elia AE, Elibol B, Filippini G, Gasser T, Krauss JK, Nardocci N, Newton A, Valls-Sole J (2011). EFNS guidelines on diagnosis and treatment of primary dystonias. Eur J Neurol.

[16] Milne I, Stephen G, Bayer M, Cock PJ, Pritchard L, Cardle L, Shaw PD, Marshall D (2013). Using Tablet for visual exploration of second-generation sequencing data. Brief Bioinform.

[17] Adzhubei I, Jordan DM, Sunyaev SR (2013). Predicting functional effect of human missense mutations using PolyPhen-2. Curr Protoc Hum Genet Chapter.

[18] Schwarz JM, Rodelsperger C, Schuelke M, Seelow D (2010). MutationTaster evaluates disease-causing potential of sequence alterations. Nat Methods.

[19] Sim NL, Kumar P, Hu J, Henikoff S, Schneider G, Ng PC (2012). SIFT web server: predicting effects of amino acid substitutions on proteins. Nucleic Acids Res.

